# A Rare Case of Sigmoid Colon Carcinoma in Incarcerated Inguinal Hernia

**DOI:** 10.3390/diagnostics10020099

**Published:** 2020-02-11

**Authors:** Dario Baldi, Vincenzo Alfano, Bruna Punzo, Liberatore Tramontano, Simona Baselice, Gianluca Spidalieri, Osvaldo Micera, Carlo Cavaliere

**Affiliations:** 1 IRCCS SDN, 80143 Naples, Italy; valfano@sdn-napoli.it (V.A.); bpunzo@sdn-napoli.it (B.P.); ltramontano@sdn-napoli.it (L.T.); sbaselice@sdn-napoli.it (S.B.); ccavaliere@sdn-napoli.it (C.C.); 2 Department of Radiology, Casa di Cura Montevergine, 83013 Mercogliano, Italy; g.spidalieri@outlook.it; 3 Section of Surgery, Santa Rita Private Care Hospital, 83042 Atripalda, Italy; osvaldomicera@gmail.com

**Keywords:** inguinal hernia, colon carcinoma, computed tomography, sigmoid colon, incarcerated

## Abstract

Incarcerated inguinal hernia is a common diagnosis in patients presenting a painful and nonreducible groin mass. Although the diagnosis is usually made by physical examination, the content of the hernia sac and the extent of the surgical operation may vary and can require multimodal imaging integration (e.g., ultrasonography, computed tomography); the usual finding is a segment of small bowel and, less commonly, large bowel. We present an extremely rare case of a sigmoid cancer incarcerated in a left inguinal hernia and infiltrating the spermatic cord. The patient underwent whole-body computed tomography (CT) with contrast agent injection for staging, followed by a left hemicolectomy paralleled by a unilateral orchiectomy.

## 1. Introduction

Inguinal hernia is a common problem in adults, with a 4% prevalence for adults >45 years of age [1]. The content of the hernial sac may vary from a piece of omentum to small or large intestine; ileum is more common than large intestine. About 10% of inguinal hernias become incarcerated, causing strangulation, bowel obstruction or infarction [2,3].

As for inguinal carcinoma, Lejars [4] classified three types of neoplasmes herniaires in 1889. When it integrates the hernial sac for a primitive pathology originating from anatomical structures, it possesses the sac such as the peritoneal serosa and the spermatic cord, such as of saccular tumors; otherwise, the pathology can affect the organ or structures organized in the sac itself, and they are named intrasaccular tumors. Extra-saccular tumors are tumors that protrude into a hernial sac but are extrinsic to the sac itself as a primitive or metastatic lymph node pathology.

Intrasaccular tumors are primary tumors of abdominal organs (for example, bladder, colon and appendix cancers) contained within the inguinal sac [5]; left colon carcinoma is the most common of these [6].

The combination of colorectal cancer and inguinal hernia is uncommon, with an estimated incidence of 0.5% in the excised sac [7]. Although the diagnosis is usually made by physical examination, the content of the hernia sac and the extent of the surgical operation may vary, and the confirmation of diagnosis can be possible only during or after surgery. Here, we highlight the computed tomography scan imaging characteristics of this rare finding in a patient’s abdominal pain. We present an 88-year-old man with a left-side sigmoid colon carcinoma in incarcerated inguinal hernia.

## 2. Case Report

Informed consent was obtained from the patient, and the local ethics committee (Comitato etico IRCCS Pascale—Naples) approved the study (project identification code: 7_19). The patient had a history of arterial hypertension, chronic obstructive pulmonary disease and a previous pulmonary embolism. He presented nonspecific abdominal pains without hernias or visible masses. For several weeks, fecal occult blood had been reported together with episodes of rectorragia, alvus alteration and episodes of diarrhea. Laboratory tests showed several abnormal values: azotaemia 70 mg/dL (normal range (nr): 10–55 mg/dL), creatinine 1.6 mg/dL (nr: 0.8–1.3 mg/dL), red blood cells 4.6 × 10^6^/µL (nr: 4.7 × 10^6^/µL to 6.1 × 10^6^/µL), hematocrit 32% (nr: 42%–52%), platelets 334 × 10^3^/µL (nr: 130 × 10^3^/µL to 400 × 10^3^/µL), neutrophils 71% (nr: 40%–68%), lymphocytes 19% (nr: 20%–45%). The tumor markers tests showed the following values: alpha-fetoprotein 2.3 U/mL (nr: 0–7 U/mL), CA125 7.5 U/mL (nr: 0–30 U/mL), CA19.9 8.9 U/mL (nr: <40 U/mL), CEA 5 ng/mL (nr: 0–2.5 or 0–3 ng/mL), troponin t high sensitivity <0.000 ng/mL (nr: <0.02 ng/mL). After laboratory tests, patient underwent whole body computed tomography (CT) and colonoscopy; whole body CT was performed on a General Electric LightSpeed 16 Slice scanner, using a standard clinical CT protocol with the following parameters: axial mode acquisition, standard filter convolution kernel, slice thickness 2.5 mm, current 365 mA, voltage 120 kV and 1.37 pitch. At first, a whole body noncontrast scan from head to feet was performed, followed by iodinated contrast agent injected into peripheral intravenous line; scan was synchronized to the injection using the prep smart technique, which consisted of placing a Region of Interest (ROI) on the descending aorta and then the injection of contrast medium automatically starting when 126 Hounsfield units were reached. Finally, a postcontrast whole body scan was achieved.

**Figure 1:**
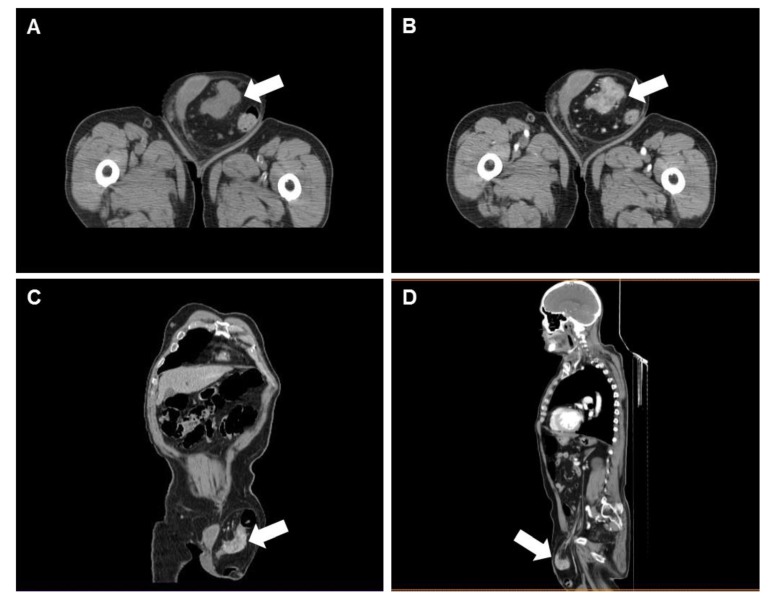
Computed tomography (CT) scan of sigmoid colon carcinoma incarcerated in inguinal hernia.: (**a**) axial plane without contrast injection; (**b**) axial plane with contrast enhancement; (**c**) coronal plane with contrast; (**d**) sagittal plane with contrast. Note: The arrows identify the pathological concentric thickening of the sigmoid wall in four different views

Whole-body CT showed a voluminous left inguinal hernia occupied by a dolichosigma loop showing thickened walls and with an intense enhancement after contrast injection (Figure 1). Other findings included cholic diverticulosis, a significant and irregular dilation of the Wirsung duct secondary to pancreatitis and a benign para-laryngeal formation.

In top row, from left to right, axial CT acquisition pre- and postcontrast injection, respectively, depict a left inguinal hernia occupied by the sigmoid tract, showing a concentric wall thickening with inhomogeneous enhancement that narrows the bowel lumen, and appearing suggestive for intrasaccular neoplasm. In bottom row, coronal and sagittal multiplanar reconstructions show the carcinoma relationship with neighboring structures. 

Colonoscopy showed a neoplastic disease 30 cm from the anus that determined a concentric stenosis of the intestinal lumen, for a length of about 8–10 cm. The lesion underwent multiple biopsies for tissue characterization and tumor grading. 

After diagnostic exams, patient underwent surgery with neuraxial anesthesia. A supraumbilical incision was performed in order to access the peritoneal cavity, where exploration showed no signs of disease repetition. Tumor in the hernial area was reached through the cavity. The testicle was intimately adhered to the organ, but there was no sign of tumor infiltration, so an inguinocrural incision was performed and the sac was isolated and then opened. Finally, after the opening of the hernial neck, the testicle and the cord attached to the sigmoid loop were also reduced into the abdominal cavity. Hemicolectomy was performed on the colic portion affected by the neoplastic mass, including the orchiectomy and an extensive mesenterectomy with conservation of nerves, ureters and vessels.

Patient had no postoperative complications, recovering his normal intestinal function in the third day postsurgery. Histological analysis was performed on the surgical specimen, showing an ulcerated-vegetative-infiltrating area of three-fourths of the bowel; the sample also included 7 cm × 3 cm of testicle with a spermatic cord 10 cm long. 

The result showed a mildly differentiated adenocarcinoma infiltrating the mucosa, submucosa, smooth muscle and subserosa, while the testicle and spermatic cord were free from neoplasia. Six pericolic lymph nodes were also excised, and a reactive lymphoreticular hyperplasia was found. The patient showed colorectal cancer stage G2 PT3 TN0, according to TNM international staging system.

After the surgical excision, the bioptic tissue was immersed in a solution of 10% neutral-buffered formalin for 24 h to preserve the cellular structure. Then, the tissue was dehydrated and embedded into paraffin blocks. Tissue sections of 4 µm were used for hematoxylin and eosin staining, with Shandon Varistain^®^ Gemini Automatic stainer (Thermo Electron Corporation), and immunohistochemistry. Rabbit monoclonal primary antibody anti-cytokeratin 7 (CK7) (SP52, catalog number: 790–4462, Roche/Ventana, Tucson, AZ, USA) and rabbit monoclonal primary antibody anti-cytokeratin 20 (CK20) (SP33, catalog number: 790–4431, Roche/Ventana, Tucson, AZ, USA) were used with OptiView DAB IHC Detection Kit on a Ventana BenchMark ULTRA automatic instrument (Roche/Ventana, Tucson, AZ, USA). The histological analysis and CK7, CK20 expressions were evaluated by a pathologist using a direct light microscope in bright field at 20× and 40× magnification. Sample analysis showed areas exhibiting histological features of adenocarcinoma, with moderately differentiated morphology, with glandular structures present in more than 50% of total tissue and submucosal invasion through the muscularis mucosa. 

**Figure 2:**
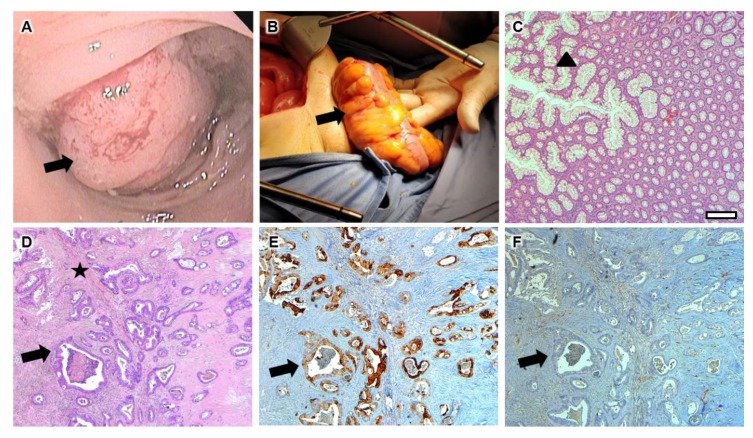
Diagnostic, interventional and immunohistochemistry workflow. (**a**) Colonoscopy view of the eroded vegetative mass (arrow) that underwent multiple biopsies; (**b**) colon excision (arrow) after both abdominal and inguinal surgical approach; (**c**) hematoxylin–eosin staining of normal sigmoid tissue (arrow-head) with numerous goblet cells; (**d**–**f**) moderately differentiated adenocarcinoma (arrow for hypercellular epithelium; star for fibrotic interstitium) stained for (**d**) hematoxylin–eosin, (**e**) CK20 and (**f**) CK7 (scale bar: 200 micrometers).

In our case, the immunohistochemistry showed a CK7-negative/CK20-positive expression pattern (Figure 2).

## 3. Discussion

A case of moderately differentiated sigmoid intrasaccular tumor was reported in an 88-year-old man that underwent physical examination, colonoscopy and whole-body CT exams.

The literature reports 38 cases of colon cancer incarcerated in hernia [3]. The first case was reported by Gerhardt and his colleagues in 1938. It is generally believed that metastatic saccular tumors are more common than intrasaccular tumors; however, the literature has found six times more intrasaccular colon cancers than saccular ones [8]. This disease was more common among elderly men and frequently involved the sigmoid colon. The inguinal hernia was predominantly in the left side. In normal conditions, sigmoidectomy with colostomy is the most frequent surgical operation, mostly without using artificial materials for the repair of inguinal hernia. Intrasaccular tumors may be herniated, as in our case, or metastasis of bowel cancer. In the case of metastatic pathology, the primary sites are the colon, stomach, pancreas, gallbladder, cutaneous melanoma, endometrium and prostate [9]; they metastasize by hematogenous route or by intracavitary dissemination, while in the case of prostate cancer, a funicular route is admitted. In the case of intrasaccular tumors, several cases are reported: omentum and mesentery sarcomas, appendix and small intestine adenocarcinomas and bladder and ovarian carcinomas; the relatively more frequent finding is adenocarcinoma of the colon, mainly on the left side [10].

The causes of high incidence are due to the relevant frequency of colorectal cancer in the population and to the equally high engagement frequency of this bowel in the hernial sac, owing to its mobile anatomy. Hypotheses that circulatory distress and the consequent inflammation of the herniated bowel were responsible for the onset of the intrasaccular tumor were formulated in the past; currently, a direct association between these two common pathologies is considered implausible [11]. No significant association between inguinal hernia and colon cancer was reported [12,13], but if there are cancer-related symptoms in patients (weight loss, rectal bleeding, abdominal pain), it is important that the radiologist considers the chance for a colon tumor in an inguinoscrotal hernia.

Most cases of inguinal hernia are usually confirmed by a physical examination, which then leads to surgery. An ultrasound can sometimes be useful for diagnosis; however, in complicated cases, as in our particular case, an ultrasound and a physical examination have a limited role in detecting its etiology, extent and environment. Main differential diagnosis was the strangulation of an irreducible inguinoscrotal hernia, mainly for intrasaccular perforated malignant tumors that can mimic clinical presentation and have a poor prognosis [14].

In our case, physical examination revealed a nonreducible inguinal groin mass, although the clinician was not able to characterize saccular content and mainly suspected oncological disease. Following, a colonoscopy was performed for the referred rectorrhagia, and a whole-body CT was performed for the unspecific oncological markers’ increase and surgical planning. A sigmoid moderately differentiated adenocarcinoma was detected within the inguinal hernial sac that circumferentially reduced colonic lumen and showed strict relationship with left spermatic cord. No signs of wall perforation, nodal involvement or metastases were detected by whole-body CT exam. Histological analyses revealed a CK7-/CK20+ phenotype, recognized as highly specific and sensitive for colorectal adenocarcinomas [15]. Indeed, while CK7 is expressed by many ductal and glandular epithelial cells (mainly gallbladder, hepatic ducts and pancreatic ducts), CK20 expression is restricted to a few organ systems, and, most importantly, almost all colon carcinomas (about 75%–95%) [16].

Regarding treatment management, for people over 85 years old with stage II colon cancer, no supportive evidence has been found for benefit from adjuvant chemotherapy, while for younger patients, higher survival rates (>16%) were reported [17,18,19].

There is also no evidence to support a certain approach in elective surgery for the inguinal carcinomas of the colon. The choice of the abdominal versus inguinal approach will depend on the patient’s anatomy, the surgical findings and the surgeon’s experience [20].

In many cases, surgery was performed in an emergency environment or carcinomas were found as incidental findings, resulting in suboptimal treatment. An extra abdominal incision was often necessary in order to perform resection, increasing the risks of complications and mortality [6,10,21,22,23].

In conclusion, the rarity of the association of inguinal hernia complicated with a tumor of the intestine must never prescind from an accurate clinical examination of the patient (even in a pathology so common as inguinal hernia) and from the search for possible symptoms relative to an equally frequent pathology such as colorectal cancer. Imaging should be carefully performed in the diagnostic workflow of this kind of patient, considering the tricky findings and the important effects on the optimal treatment planning.
